# Advanced Robust Heading Control for Unmanned Surface Vessels Using Hybrid Metaheuristic-Optimized Variable Universe Fuzzy PID with Enhanced Smith Predictor

**DOI:** 10.3390/biomimetics10090611

**Published:** 2025-09-10

**Authors:** Siyu Zhan, Qiang Liu, Zhao Zhao, Shen’ao Zhang, Yaning Xu

**Affiliations:** 1 Department of Electrical Engineering, Baltic State Technical University, 190000 St. Petersburg, Russia; e5m0109@voenmeh.ru; 2 School of Electronic Engineering, Jiangsu Ocean University, Lianyungang 222005, China; 2008000034@jou.edu.cn; 3 China Merchants Jinling Shipbuilding (Jiangsu) Co., Ltd., Yizheng 211400, China; 4 School of Naval Architecture and Ocean Engineering, Jiangsu Ocean University, Lianyungang 222005, China; 2024221711@jou.edu.cn (S.Z.); 2024221709@jou.edu.cn (Y.X.)

**Keywords:** unmanned surface vessels (USVs), heading control, variable-universe fuzzy PID, smith predictor, hybrid metaheuristic optimization, robust control, time-delay compensation, marine autonomy, adaptive control, intelligent optimization

## Abstract

With the increasing deployment of unmanned surface vessels (USVs) in complex marine operations such as ocean monitoring, search and rescue, and military reconnaissance, precise heading control under environmental disturbances and system delays has become a critical challenge. This paper presents an advanced robust heading control strategy for USVs operating under these demanding conditions. The proposed approach integrates three key innovations: (1) an enhanced Smith predictor for accurate time-delay compensation, (2) a variable-universe fuzzy PID controller with self-adaptive scaling domains that dynamically adjust to error magnitude and rate of change, and (3) a hybrid metaheuristic optimization algorithm combining beetle antennae search, harmony search, and genetic algorithm (BAS-HSA-GA) for optimal parameter tuning. Through comprehensive simulations using a Nomoto first-order time-delay model under combined white noise and second-order wave disturbances, the system demonstrates superior performance with over 90% reduction in steady-state heading error and ≈30% faster settling time compared to conventional PID and single-optimization fuzzy PID methods. Field trials under sea-state 4 conditions confirm 15–25% lower tracking error in realistic operating scenarios. The controller’s stability is rigorously verified through Lyapunov analysis, while comparative studies show significant improvements in S-shaped path tracking performance, achieving better IAE/ITAE metrics than DRL, ANFC, and ACO approaches. This work provides a comprehensive solution for high-precision, delay-resilient USV heading control in dynamic marine environments.

## 1. Introduction

With the development of autonomous navigation technology, unmanned surface vehicles (USVs) are increasingly employed in ocean monitoring, military reconnaissance, search and rescue, etc. [1]. Typically operating in complex and dynamic marine environments, USVs require motion control systems with high robustness and adaptability to address challenges such as environmental disturbances, nonlinear hull dynamics, and inherent system delays. In response to the USV heading control problem, researchers have proposed a variety of control methods, including proportional-integral-derivative (PID) control, fuzzy control, sliding mode control (SMC), model predictive control (MPC) and intelligent control methods based on neural networks [2,3]. Among them, traditional PID control remains widely adopted in USV heading control due to its simple structure and easy implementation [4]. However, it exhibits limited performance in nonlinear, coupled, and time-delay systems, leading to reduced control precision and degraded stability.

Time-delay compensation plays a crucial role. The work in Reference 6 demonstrates that even enhanced SP variants introduce prediction mismatches and weakened robustness when faced with model-plant mismatches. Recent developments have attempted to enhance SP adaptability through adaptive or data-driven modifications and integration with intelligent controllers [5]. Nonetheless, these approaches often struggle to maintain a balance between compensation accuracy and real-time robustness in highly dynamic environments [6].

Fuzzy PID control has been used for nonlinear heading control of USV due to its strong adaptability. However, the domain of traditional fuzzy PID controllers is usually fixed, making it difficult to adapt to error changes under different working conditions. Variable universe fuzzy control (VUFC) can adaptively adjust the input and output universe according to the error size, thereby improving the flexibility and response speed of the control system [7]. Studies have shown that variable universe fuzzy PID control exhibits better dynamic performance in nonlinear and time-varying systems [8]. Despite these advantages, the selection of its control parameters still relies heavily on empirical adjustment and trial-and-error methods, making it difficult to achieve global optimization and often resulting in sub-optimal performance. Create a table summarizing the literature review as shown in Table 1, introducing the problems and solutions of the relevant literature research content.

Therefore, a significant research gap persists for a unified control strategy that can simultaneously overcome three intertwined challenges: (1) the robustness issue of time-delay compensators under modeling uncertainties and environmental disturbances; (2) the sub-optimal performance of adaptive controllers due to empirical parameter tuning methods; and (3) the lack of a global optimization framework capable of efficiently handling the complex parameter space of sophisticated controllers like VUFC under real-world marine conditions. To bridge this gap, this work develops a novel delay-tolerant control architecture that integrates three key innovations: First, an enhanced Smith predictor structure for accurate time-delay compensation under modeling uncertainties. Second, a variable-universe fuzzy PID controller with self-adaptive scaling domains that dynamically adjust to error magnitude and rate of change. Third, a hybrid metaheuristic optimization algorithm combining beetle antennae search, harmony search, and genetic algorithm (BAS-HSA-GA) for global optimal parameter tuning.

Following this introduction, the remainder of this paper is organized as follows: Section 2 establishes the theoretical background, detailing the principles of variable universe fuzzy control and the Smith predictor. Section 3 presents the design and optimization of our proposed heading control system, including the USV model, controller structure, stability analysis, and the hybrid BAS-HSA-GA optimization algorithm. Section 4 provides a comprehensive simulation analysis, comparing system performance under various conditions and against different algorithms. And describes field validation tests conducted under realistic marine conditions. Finally, Section 5 concludes the paper with a summary of key findings and suggestions for future research directions.

## 2. Theoretical Background

### 2.1. Basic Concepts of Variable Universe Fuzzy Control

**Figure 1:**
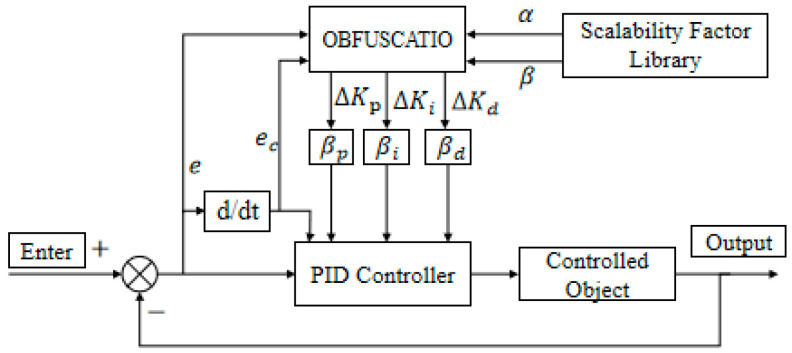
Structural Layout of a Variable-Domain Fuzzy PID Controller.

Variable universe fuzzy control integrates the concept of variable universes into fuzzy control systems without altering the original fuzzy rule base [9,10]. Instead, it dynamically adjusts the input and output domain ranges according to input variations by applying specific scaling rules. Classical fuzzy control systems with fixed domains often struggle to maintain optimal control performance under external disturbances [11]. In contrast, variable universe fuzzy control adapts the domain size based on the magnitude of the input: the domain contracts when the input is small and expands when the input grows. This adaptive domain adjustment effectively mitigates system oscillations and enhances anti-disturbance capabilities, thereby improving overall control performance. Figure 1 illustrates the structural layout of a variable-universe fuzzy PID controller [12,13].

Suppose the system error falls within [−E1,E1], and the initial fuzzy rule set includes NS, ZO, and PS. After domain compression, the rule set expands to NM, NS, ZO, PS, and PM, resulting in finer fuzzy partitions and enhanced control precision. Although the domain is compressed, the number of fuzzy rules does not increase, so the impact on the system response speed is small [14]. This method ensures that while improving the control accuracy, it can still maintain a faster response speed.

(1) $$\left\{\begin{array}{c} U_{x}\left(t\right)=k_{x}\left(t\right)\cdot U_{x0},\\ U_{y}\left(t\right)=k_{y}(t)\cdot U_{y0} \end{array}\right.$$

Let $U_{x0}$ and $U_{y0}\;$ denote the initial input and output domains, respectively. The dynamically adjusted scaling factors $k_{x}\left(t\right)\;$ and $k_{y}(t)$, which vary with the error, are given by Equations (2) and (3).

(2) $$k_{x}\left(t\right)=1+\alpha_{x}\cdot {\left(\frac{\left|e\left(t\right)\right|}{E_{max}}\right)}^{\beta },$$

(3) $$k_{y}\left(t\right)=1+\alpha_{y}\cdot {\left(\frac{\left|e\left(t\right)\right|}{E_{max}}\right)}^{\gamma }$$

Here, $\alpha_{x}$ and $\alpha_{y}$ are tuning coefficients, β and  γ are exponents for the nonlinear transformation, and $E_{max}$ represents the maximum error.

#### 2.1.1. Contraction and Expansion of Basic Domain

**Figure 2:**
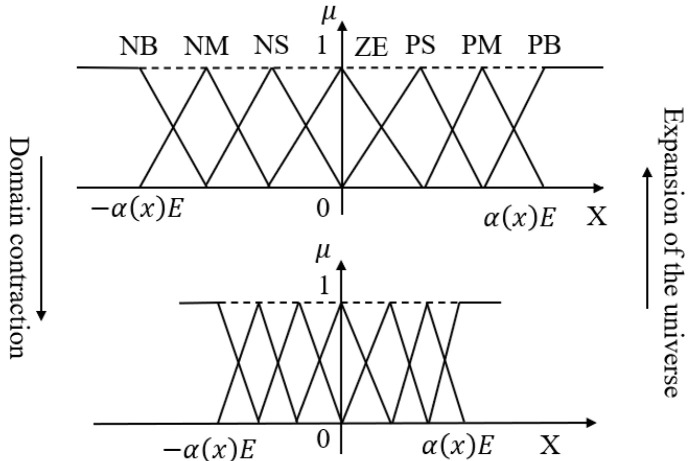
The scaling transformation diagram of the domain.

Variable universe fuzzy control adjusts the control effect by contracting or expanding the basic domain, rather than directly scaling the fuzzy domain [1]. Figure 2 depicts the scaling transformation of the domain.

For contraction, if the original basic domain is [−e,e], after contraction, it becomes $\left[-e_{1},e_{1}\right]$, with the scaling factor $a=e/e_{1}$ lying in [0,1], The transformations are given by (4) and (5) [15]:

(4) $$\left[-\alpha E,\alpha E\right]=\left[-\alpha e,\alpha e\right]\cdot \frac{E}{e}=\left[-e_{1},e_{1}\right]\cdot \frac{E}{e}$$

(5) $$\left[-E,E\right]=\left[-e,e\right]\cdot \frac{E}{e}=\left[-\frac{e_{1}}{\alpha },\frac{e_{1}}{\alpha }\right]\cdot \frac{E}{e}$$

The actual input $x_{1}$ to the fuzzy controller, after domain contraction to $\left[-e_{1},e_{1}\right]$, falls within  [−αE,αE]. The transformation for the input x is expressed as:

(6) $$x=\left(\frac{x}{\alpha }\right)\cdot \left(\frac{E}{e}\right)$$

Here, $\frac{x}{\alpha }$ converts the contracted domain $\left[-e_{1},e_{1}\right]$ back to the original basic domain [−e,e], while $\frac{E}{e}$ scales the basic domain to the fuzzy domain [−E,E]. This method maintains the fuzzy domain unchanged while effectively amplifying the input and output through basic domain adjustment [16].

For expansion, when [−e,e] extends to $\left[-e_{2},e_{2}\right]$, the scaling factor $a=e/e_{2}$ again lies in [0,1]. The relationship between the expanded basic domain and the fuzzy domain is given by Formulas (7) and (8). This processing method allows for the expansion and contraction of the basic domain to adapt the input and output values while leaving the fuzzy domain unchanged [17].

(7) $$\left[-\frac{E}{\alpha },\frac{E}{\alpha }\right]=\left[-\frac{e}{\alpha },\frac{e}{\alpha }\right]\cdot \frac{E}{e}=\left[-e_{2},e_{2}\right]\cdot \frac{E}{e}$$

(8) $$\left[-E,E\right]=\left[-e,e\right]\cdot \frac{E}{e}=\left[-\alpha e_{2},\alpha e_{2}\right]\cdot \frac{E}{e}$$

#### 2.1.2. Introduction of Scaling Factors

The introduction of the scaling factor α(t) forms the foundation of variable universe adjustment. It dynamically modifies the input and output domain sizes based on the real-time error value [18]. This approach enhances system adaptability and mitigates instability caused by abrupt error variations. Its computation is described by:

(9) $$\alpha (t)=\frac{1}{1+\gamma \cdot {\left|e(t)\right|}^{p}}$$

where γ is the adjustment factor and p is the power exponent. When the error e(t) is large, the scaling factor α(t) is close to zero, which expands the domain; when the error is small, the scaling factor is close to 1, which shrinks the domain. When addressing the time variation factor, the time constant τ can be introduced as illustrated in Formula (10):

(10) $$\alpha \left(t\right)=\frac{1}{1+\gamma \cdot {\left|e\left(t\right)\right|}^{p}+\frac{t}{\tau }}$$

This permits the domain adjustment to account for both the error and the time effect.

### 2.2. Principles of Smith Predictor

The Smith predictor is a classical control method designed for systems with time delay, a common challenge in unmanned surface vehicle (USV) heading control due to sensor transmission and actuator response lags [19].

**Figure 3:**
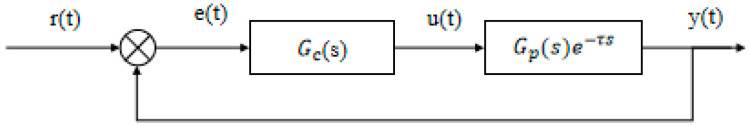
Control system with pure lag link.

As shown in Figure 3, the closed-loop control system includes a time delay term $e^{\tau s}$, indicating that the signal is delayed by τ seconds without altering its characteristics [20]. This delay hinders the controller’s ability to respond promptly to external disturbances, potentially leading to overshoot and oscillation, thereby affecting system stability. Unlike hysteresis, however, $e^{\tau s}$ represents only a fixed delay and does not introduce additional dependencies on past input values beyond τ.

**Figure 4:**
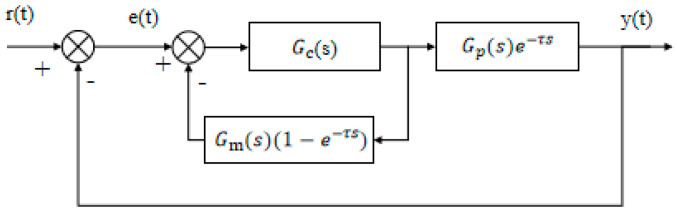
Smith estimation principle diagram.

The core concept of the Smith predictor, shown in Figure 4, is to use a model-based prediction to compensate for the delay’s adverse effects. By utilizing a known delay model and current system state, the predictor forecasts future system outputs to enable real-time control despite the delay, substantially enhancing accuracy and response speed [21].

Where $G_{c}(s)$ is the transfer function of the controller, $G_{p}(s)$ is the transfer function of the controlled object after removing the time lag, τ is the time lag factor of the controlled object, $G_{m}(s)$ is the transfer function of the estimated model after removing the time lag [15], $\tau_{m}$ is the time lag factor of the estimated model, r(s) is the system input, and y(s) is the system output [22]. When the controlled object and the estimated model are completely matched, that is, $G_{p}(s)=G_{m}(s)$ and $\tau =\tau_{m}$, the closed-loop transfer function composed of the controller and the Smith predictor is (11) [23,24]:

(11) $$W(s)=\frac{G_{c}(s)}{1+G_{c}(s)G_{p}(s)}$$

The closed-loop transfer function of the compensated system is (12):

(12) $$\phi \left(s\right)=\frac{G\left(s\right)}{1+W\left(s\right)G\left(s\right)}+\frac{G_{c}\left(s\right)G_{p}\left(s\right)}{1+G_{c}\left(s\right)G_{p}\left(s\right)}$$

After applying the Smith predictive control technique, the closed-loop transfer function φ(s) has the following characteristic Equation (13):

(13) $$1+G_{c}\left(s\right)G_{p}\left(s\right)=0$$

This indicates that the time delay term $e^{-\tau s}$ has been compensated.

For the unmanned surface vehicle (USV), the heading dynamics are often simplified using a first-order time-delay system to capture the main dynamic characteristics of the vehicle’s response. This simplification is based on the assumption that the USV’s maneuvering dynamics can be accurately represented with a low-order model, where the influence of higher-order terms is relatively small. A first-order model with time delay is used to capture the sensor measurement delay and actuator response delay in the control loop. In this study, the heading transfer function of the USV is modeled as (14):

(14) $$G_{\psi }(s)=\frac{K_{\psi }}{T_{\psi }s+1}e^{-\tau_{\psi }s}$$

where $K_{\psi }$ is the heading gain, $T_{\psi }$ is the time constant, and $\tau_{\psi }$ is the time lag [12], which are determined through system identification methods or experimental data from the USV. This model effectively accounts for the time delays inherent in the system, allowing for accurate control performance with time delay compensation via the Smith predictor. Through the Smith predictor, the output of the system can be predicted as (15) [25]:

(15) $${\hat{y}}_{\psi }\left(t+\tau_{\psi }\right)=G_{0}(s)\cdot u(t)$$

## 3. Design and Optimization of Heading Control System

### 3.1. Basic Principles of the Control System

The proposed heading control system integrates three fundamental components to achieve robust performance in challenging marine environments. First, a variable universe fuzzy PID controller forms the core control logic, which dynamically adjusts its operational domains based on the magnitude of tracking error and its rate of change. This adaptive mechanism enables finer control precision when errors are small and broader authority when errors are large. Second, an enhanced Smith predictor compensates for inherent system time delays by forecasting future system states and providing advance compensation signals. This component effectively removes the destabilizing effects of time delays from the control loop. Third, a hybrid optimization algorithm combines the strengths of beetle antennae search, harmony search, and genetic algorithms to automatically determine the optimal parameters for both the fuzzy controller and Smith predictor. This triple-layered approach ensures precise heading control despite environmental disturbances, system nonlinearities, and sensor-actuator delays that characterize unmanned surface vehicle operations.

### 3.2. Unmanned Ship Model

To evaluate the performance of various heading control strategies, simulation experiments were conducted using Simulink on the MATLAB(R2022a) platform. The model parameters for the ‘Lanxin’ unmanned surface vehicle (USV) were adopted from the well-established benchmark model developed by Dalian Maritime University [26]. This platform was chosen for its strong capabilities in modeling complex nonlinear control systems, particularly its excellent support for high-fidelity modeling of hybrid continuous/discrete systems, inherent time delays, and external environmental disturbances (e.g., waves and wind). Its status as an industry standard also ensures the reproducibility and reliability of the simulation results. The heading dynamics are modeled using the first-order Nomoto model with a time delay, characterized by parameters K=0.71 and T=0.32, and $\tau_{\psi }=0.8$, identified through empirical data. The key physical parameters of the USV are summarized in Table 2. These parameters are used in the time-delay system model for the USV’s heading dynamics, Equation (14).

The simulation spans 100 s with a sampling interval of 0.1 s. Considering the environmental disturbances typically encountered in real marine operations, such as wind, waves, and currents [27], the simulation incorporates interference modeled by white Gaussian noise and a second-order wave transfer function as given in Equation (16) [28]:

(16) $$Y_{d}\left(s\right)=h\left(s\right)\omega (s)$$

Here, $\omega \left(s\right)\;$ represents zero-mean Gaussian white noise, and h(s) is defined as: (17) [29]:

(17) $$\left\{\begin{array}{l} h\left(s\right)=\frac{K_{\omega }s}{s^{2}+2\zeta \omega_{0}s+{\omega_{0}}^{2}}\\ K_{\omega }=2\zeta \omega_{0}\sigma_{w}\\ \begin{array}{l} \omega_{0}=\frac{4.85}{T_{w}}\\ \sigma_{w}=\sqrt{0.0185T_{w}h_{1/3}} \end{array} \end{array}\right.$$

For a sea state corresponding to Beaufort scale level 4, the parameters are set as: $T_{w}$ is taken as 5 s, $h_{1/3}=1.25\;m$, ζ=0.2, resulting in the specific wave model (18):

(18) $$h\left(s\right)=\frac{0.14744s}{s^{2}+0.3776s+0.9409}$$

### 3.3. Design of Fuzzy Control with Variable Universe

#### 3.3.1. Fuzzy PID Controller Design

**Figure 5:**
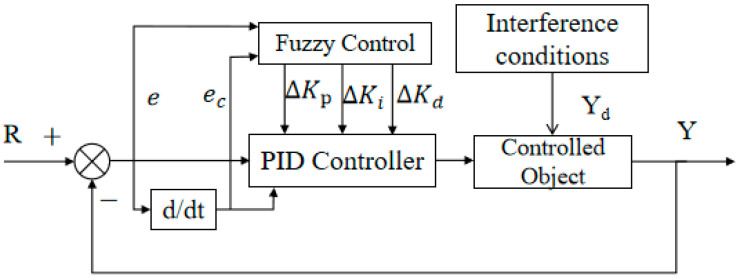
Architecture of the fuzzy PID control system.

To enhance heading control performance, a fuzzy PID controller that integrates conventional PID control and fuzzy logic reasoning is developed. The design involves the definition of fuzzy subsets, their corresponding universes of discourse and membership functions, the establishment of fuzzy control rules, and the incorporation of online self-tuning strategies based on accumulated operational knowledge, as illustrated in Figure 5 [30].

#### 3.3.2. Controller Structure

Building upon the classical fuzzy PID framework, the proposed controller introduces a domain adaptation mechanism that adjusts the universe of discourse in real-time based on input signals [31]. This adaptive scaling mechanism improves control performance by enabling fine-grained tuning of parameters without altering the basic domain structure.

#### 3.3.3. Design and Implementation of Fuzzy Rules

A dual-input, single-output fuzzy controller with scalable coefficients is adopted [30,32]. The input variables of the controller are error and error change rate, and the output variables include error expansion factor $\alpha_{1}$, error change rate expansion factor $\alpha_{2}$, and expansion factor $\beta_{1}$ of proportional action adjustment $∆K_{p}$ and expansion factor $\beta_{2}$ of integral action adjustment $∆K_{i}$.

**Figure 6:**
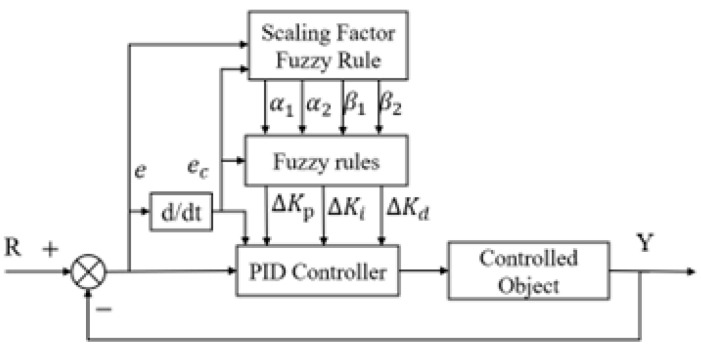
Schematic diagram of dual-input single-output variable domain heading control structure.

Each output depends solely on its corresponding input, justifying the “dual-input, single-output” naming convention. The dual-input single-output structure shown in Figure 6 builds upon the conventional fuzzy PID controller architecture [29–31] by introducing variable domain adaptation mechanisms.

Fuzzy variables are typically categorized as “NB, NM, NS, ZO, PS, PM, PB.” The output variables $\alpha_{1}$ and $\alpha_{2}$ of the controller have a domain range of [0,1] and are classified into “B, M, S, ZO” [14].

In the dual-input single-output fuzzy PID controller, the error scaling factor $\alpha_{1}$ is proportional to the error, while the error change rate scaling factor $\alpha_{2}$ corresponds to the error change rate [33].

The rules for $\alpha_{1}$ and $\alpha_{2}$ are simpler due to their direct correlation with error and error rate [30]. In contrast, $\beta_{1}$ and $\beta_{2}$ depend on the joint variation in both, making rule design more complex [34].

### 3.4. Improved Design of Smith Predictor

**Figure 7:**
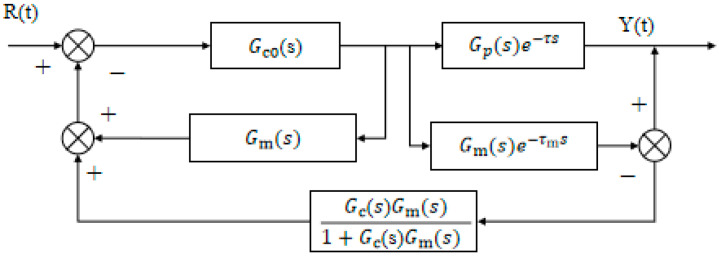
C.C.Hang Smith estimation model.

The improved Smith predictor proposed by C.C. Hang significantly enhances the adaptability of classical Smith structures in systems with time delay [35]. The Smith estimation model depicted in Figure 7 follows the structure proposed by Hang et al. [22] and implemented according to [23].

Among them, $G_{co}(S)$ and $G_{s}(S)$ represent the transfer functions of the main regulator and the auxiliary regulator, respectively. The feedback loop gain of the traditional Smith predictor is 1 [23], while the improved feedback loop adopts the $G_{f}(S)$ algorithm structure, and the expression is shown in Formula (19):

(19) $$G_{f}(s)=\frac{G_{c}(s)G_{m}(s)}{1+G_{c}(s)G_{m}(s)}$$

**Figure 8:**
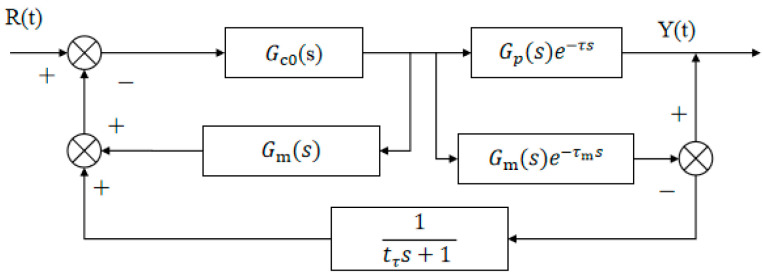
Reduced schematic of the adaptive Smith predictor with improvements.

When $G_{s}(S)$ is used as a PI regulator and $G_{m}(s)$ is a first-order inertia gain, according to the control principle, the integral time of the regulator is equivalent to the model time, so $G_{f}(S)$ is simplified to Formulas (20) and (21):

When $G_{s}(S)$ (s) functions as a PI controller and $G_{m}(s)$ represents a first-order inertial gain, the regulator’s integral time matches the model time, allowing $G_{f}(S)$ to be simplified as shown in Equations (20) and (21).

(20) $$G_{f}\left(S\right)=\frac{1}{\frac{t_{m}}{k_{c}k_{m}}s+1}=\frac{1}{t_{f}s+1}$$

(21) $$t_{f}s=\frac{t_{m}}{k_{c}k_{m}}$$

where $t_{f}$ represents the excitation time constant of the system, $t_{m}$ represents the time constant of the Smith predictor, $k_{c}$ is the gain of $G_{s}(S)$, and $k_{m}$ is the gain of the Smith predictor. The simplified schematic in Figure 8 maintains the core compensation principle of the Smith predictor [22] while incorporating our enhancements for improved adaptability.

By applying modern control theory to derive the closed-loop function, the simplified model’s closed-loop transfer function is obtained as Equation (22) [23]:

(22) $$\frac{Y\left(s\right)}{R\left(s\right)}=\frac{G_{c}\left(s\right)G_{p}\left(s\right)e^{-\tau s}}{1+G_{c}\left(s\right)G_{m}\left(s\right)+G_{c}\left(s\right){(G}_{p}\left(s\right)e^{-\tau s}-G_{m}\left(s\right)e^{-\tau_{m}s})\frac{1}{t_{f}s+1}}$$

From the above formula, we can see that when the model is completely matched, that is (23) and (24);

(23) $$G_{m}\left(s\right)=G_{p}(s)$$

(24) $$\tau =\tau_{m}$$

At this time, the transfer function can be changed to (25):

(25) $$\frac{Y(s)}{R(s)}=\frac{G_{c}(s)G_{p}(s)e^{-\tau s}}{1+G_{c}\left(s\right)G_{p}\left(s\right)}$$

From the transfer function of Equation (25), it can be seen that the lag term $e^{-\tau s}$ in the closed-loop transfer function has been removed from the system, so the closed-loop system has better control performance. In addition, the first-order inertia link $\frac{1}{t_{f}s+1}$ has no effect on the system. When the Smith predictor deviates from the controlled object, the following events may occur (26) and (27).

(26) $$G_{m}\left(s\right)\neq G_{p}\left(s\right),\tau \neq \tau_{m}$$

(27) $$G_{m}\left(s\right)\neq G_{p}\left(s\right)\&\tau \neq \tau_{m}$$

### 3.5. Lyapunov Stability Analysis

#### 3.5.1. Overview of Stability Analysis

In this study, we use Lyapunov stability theory to perform stability analysis on the designed fuzzy PID controller [36]. This method can verify whether the designed controller can ensure the stability of the system when facing external disturbances and system dynamic changes.

#### 3.5.2. Construction of Lyapunov Function

In order to perform Lyapunov stability analysis, we first define the state variables of the system. Considering the heading error $e_{\psi }(t)$ and the derivative of the heading error $\dot{e_{\psi }}\left(t\right)$, we construct the following Lyapunov function (28):

(28) $$V\left(e_{\psi },\dot{e_{\psi }}\right)=\frac{1}{2}{e_{\psi }}^{2}+\frac{1}{2}{\dot{e_{\psi }}}^{2}$$

where $e_{\psi }$ represents the heading error and $\dot{e_{\psi }}$ is the derivative of the heading error.

#### 3.5.3. Derivation of Lyapunov Function

To ascertain the system’s stability, it is essential to examine the rate of change in the Lyapunov function over time. Based on the control system’s dynamic equations, we derive the temporal changes in both the heading error and its rate of change as follows (29) and (30):

(29) $$\dot{e_{\psi }}\left(t\right)=\psi_{desired}\left(t\right)-\psi \left(t\right)$$

(30) $$\ddot{e_{\psi }}\left(t\right)={\dot{\psi }}_{desired}\left(t\right)-\dot{\psi }\left(t\right)$$

Under the action of the fuzzy PID controller, the control input *u*(*t*) of the system can be expressed as (31) [37]:

(31) $$u\left(t\right)=K_{p}\cdot e_{\psi }\left(t\right)+K_{i}\int e_{\psi }\left(t\right)dt+K_{d}\cdot \dot{e_{\psi }}\left(t\right)$$

This control input is used to correct the heading error $e_{\psi }\left(t\right)$ so that the system can be stabilized.

The time derivative of the Lyapunov function $\dot{V}\left(e_{\psi },\dot{e_{\psi }}\right)$ can be obtained by differentiating $V\left(e_{\psi },\dot{e_{\psi }}\right)$.

We use the chain rule to differentiate $V\left(e_{\psi },\dot{e_{\psi }}\right)$ and obtain (32):

(32) $$\dot{V}\left(e_{\psi },\dot{e_{\psi }}\right)=e_{\psi }\cdot \dot{e_{\psi }}+\dot{e_{\psi }}\cdot \ddot{e_{\psi }}$$

Substituting into the controller dynamic Equation (33):

(33) $$\dot{V}\left(e_{\psi },\dot{e_{\psi }}\right)=e_{\psi }\cdot \dot{e_{\psi }}+\dot{e_{\psi }}\cdot (-K_{p}e_{\psi }-K_{i}\int e_{\psi }\left(t\right)dt-K_{d}\dot{e_{\psi }})$$

After simplification, we receive (34):

(34) $$\dot{V}\left(e_{\psi },\dot{e_{\psi }}\right)=-K_{p}{e_{\psi }}^{2}-K_{d}{\dot{e_{\psi }}}^{2}-K_{i}e_{\psi }\int e_{\psi }\left(t\right)dt$$

#### 3.5.4. Stability Conditions

To ensure the stability of the system, we need to ensure that $\dot{V}\left(e_{\psi },\dot{e_{\psi }}\right)$ is always non-positive [22]. By analyzing the role of controller gains $K_{p}$, $K_{i}$, and $K_{d}$, we can derive the following conditions:

When $K_{p}>0$, $K_{d}>0$, $K_{i}\geq 0$, $\dot{V}\left(e_{\psi },\dot{e_{\psi }}\right)$ will always be negative or zero, thus ensuring the stability of the system [10].

If $K_{i}>0$, the integral action helps to eliminate the steady-state error of the system, thereby improving the accuracy of the system.

Therefore, since the gain of the fuzzy PID controller design is selected as $K_{p}=35$, $K_{d}=1$, $K_{i}=100$, it can ensure that the system remains stable in the face of disturbances [13,38].

### 3.6. Parameter Optimization Design Based on BAS-HSA-GA Hybrid Optimization Algorithm

To further improve controller performance, a hybrid optimization algorithm is developed by integrating the Beetle Antennae Search (BAS), Harmony Search Algorithm (HSA), and Genetic Algorithm (GA). This hybrid algorithm combines global exploration and local refinement, improving convergence speed and robustness.

#### 3.6.1. The Main Steps of Algorithm Implementation

Step 1: Initialization phase.

Determine the optimization goal and problem dimension: Let the optimization problem be to minimize the function f(x), where $x=(x_{1},x_{2},{\ldots ,x}_{D})$ is a D-dimensional vector representing the solution to the problem. f(x) is the objective function defined by the optimization problem. Set the parameter dimension D = 5 (corresponding to $K_{P},K_{i},K_{d},K_{e},K_{ec}$).

Population size and subpopulation division: Determine the size N of the entire population, BAS, HSA and GA subpopulations, $N=N_{BAS}+N_{HSA}+N_{GA}=10$.

Algorithm parameter settings: beetle whisker length $d_{0}=1$; perception distance coefficient k=0.5; parameter upper and lower bounds Ub=[100,100,10,10,10], Lb=[0,0,0,0,0]; harmony memory size HMS, determines the number of optimal solutions that can be stored in the harmony memory, $HMS\leq N_{HSA}$, set HMS=10; harmony memory consideration rate HMCR=0.9; pitch adjustment rate PAR=0.3.

Population initialization: Initialize the individual position $x_{ij}^{0}(i=1,2,\ldots ,N;j=1,2,\ldots ,D)$, $x_{ij}^{0}\in [a_{j},b_{j}],[a_{j},b_{j}]$ is the j-th dimension of the search space; calculate the initial fitness value $f(x_{i}^{0})$ of each individual and convert it into a fitness value using Formula (35):

(35) $$f(x_{i}^{0})=1/(1+f(x_{i}^{0}))$$

Step 2: Algorithm iteration

1. Iteration process of BAS algorithm

Direction vector generation: For individuals $x_{i}^{t}(i=\;1,2,\ldots ,n_{1})$ in the BAS subpopulation, randomly generate a unit direction vector (36):

(36) $$\vec{d}=(d_{1},d_{2},\ldots ,d_{D})$$

Each element $d_{j}\in [-1,1]$ satisfies $\sum_{j=1}^{D}d_{j}^{2}=1$.

Calculation of the position of the beetle whiskers: Based on the current individual position $x_{i}^{t}$, the length of the beetle whiskers $d_{0}$, and the direction vector $\vec{d}$, calculate the left whisker position $x_{iL}^{t}$ and the right whisker position $x_{iR}^{t}$, left and right whisker position (37):

(37) $$x_{iL}^{t}=x_{i}^{t}-\frac{d_{0}}{2}\vec{d},\;\;x_{iR}^{t}=x_{i}^{t}+\frac{d_{0}}{2}\vec{d}$$

Fitness evaluation and update: Calculate the left and right whisker positions $x_{iL}^{t}$ and $x_{iR}^{t}$, fitness values $f(x_{iL}^{t})$ and $f(x_{iR}^{t})$, and use the predefined objective function f(x) for calculation. If $f(x_{iL}^{t})>f(x_{iR}^{t})$, then $x_{i}^{t+1}=x_{iL}^{t}$. Otherwise, update to $x_{i}^{t+1}=x_{iR}^{t}$.

Step size update: Update the length of the longhorn beetle whisker, Formula (38):

(38) $$d_{0}^{t+1}=d_{0}^{t}\times 0.95$$

where α is the contraction coefficient, usually α∈(0,1) to gradually reduce the step size.

2. HAS algorithm operation process

Initialize the harmony memory bank, select HMS individuals to form the harmony memory bank HM, and record the fitness value corresponding to each individual (39) [39]:

(39) $${\left\{f(x_{h}^{0})\right\}}_{h=1}^{HMS}$$

Generate a new solution. For each iteration t, generate a new solution (40):

(40) $$x_{new}^{t}=(x_{new1}^{t},x_{new2}^{t},\ldots ,x_{newD}^{t})$$

The generation rule is as follows: For each dimension j= 1,2,…,D, with probability (41):

(41) $$\mathrm{HMCR}\;:x_{newj}^{t}=x_{hj}^{t}$$

where h is an index randomly selected from 1 to HMS. Randomly generated with probability $\left(1-HMCR\right):x_{newj}^{t}\in [a_{j},b_{j}]$. Use the Levy flight mechanism to integrate pitch adjustment: Generate a Levy flight step vector $L=(L_{1},L_{2},\ldots ,p)$, whose element $L_{j}$ follows the Levy distribution. Use the generated Levy flight step to adjust the new solution. The new solution adjustment Formula (42) is:

(42) $$x_{newj}^{t}=x_{newj}^{t}+L_{j}\times bw_{j}\times r,(j=1,2,\ldots ,D)$$

where $bw_{j}$ is the bandwidth (adaptively adjusted according to factors such as the number of iterations), which is used to control the amplitude of Levy flight adjustment, and r∈[0,1] is a random number.

Fitness evaluation and update: Calculate the fitness of the new solution $x_{newj}^{t}$ as $f(x_{newj}^{t})$, and calculate it with the previous objective function f(x) to find the worst individual setting index $h_{min}$ in the harmony memory library.

If $f(x_{newj}^{t})>f(x_{h_{min}}^{t})$, replace the worst solution with the new solution, that is (43):

(43) $$x_{h_{min}}^{t}=x_{newj}^{t},\;\;f\left(x_{newj}^{t}\right)=f(x_{h_{min}}^{t})$$

3. GA subpopulation operation process

First, the selection operation: for $\left(i=1,2,\ldots ,n_{3}\right)$, calculate the selection probability (44):

(44) $$p_{i}^{t}=\frac{f\left(x_{i}^{t}\right)}{\sum_{k=1}^{n_{3}}f\left(x_{k}^{t}\right)}$$

Use the roulette method to select parent individuals. Individuals with higher fitness have a greater probability of being selected

The crossover operation creates new offspring by recombining the genes of two parent individuals. In this algorithm, it is performed by randomly selecting two parents, $x_{p1}$ and $x_{p2}$, from the selected population, and generating two offspring through recombination.

Mutation operation: For each individual in the population, a mutation operation is performed with a probability of $P_{m}$. For the selected individual, a dimension j is randomly selected and perturbed in a small range to ensure that the mutated individual is still within the upper and lower bounds of the parameter.

The individual $x_{ij}^{t}(i=1,2,..,n_{3};j=1,2,\ldots ,D)$ is mutated with probability $P_{m}$, that is, Formula (45):

(45) $$x_{ij}^{t+1}=x_{ij}^{t}+∆x_{ij}$$

where $∆x_{ij}=\sigma \times r$ (σ is the variable step length, r∈[0,1] random number).

Step 3: Termination condition judgment

When $t=T_{max}$ or the fitness value is less than the threshold, the algorithm is terminated and the individual with the highest fitness in the entire population is selected as the final solution [40].

Use the collaborative mechanism to migrate the optimal solution between subgroups every 10 generations to avoid premature convergence. Update the global optimal solution (46):

(46) $$x_{ij}^{t+1}=x_{ij}^{t}+∆x_{ij}$$

Optimize the objective function and minimize the ITSE index (47):

(47) $$ITSE=\int_{0}^{T}t\cdot e^{2}\left(x\right)dt$$

Step 4: Output the optimal parameters, $gBest=[K_{P},K_{i},K_{d},K_{e},K_{ec}]$.

#### 3.6.2. Dynamic Upper and Lower Bound Adjustment Mechanism

To improve search adaptability, a dynamic bound adjustment mechanism is introduced based on changes in the optimal solution. If no significant improvement occurs over a set number of iterations, the algorithm automatically modifies the upper and lower bounds to either expand or shrink the search space [41]. The corresponding adjustment expressions are given in Formulas (48) and (49).

(48) $$Ub\left(t+1\right)=z_{best}+\frac{Ub\left(t\right)-Lb\left(t\right)}{\alpha }$$

(49) $$Lb\left(t+1\right)=z_{best}-\frac{Ub\left(t\right)-Lb\left(t\right)}{\alpha }$$

Here, α is the adjustment coefficient, and $z_{best}$ represents the current global optimum, these enhancements significantly improve the global search capability and convergence speed of the proposed hybrid algorithm in optimizing fuzzy PID controller parameters [11]. Additionally, incorporating mechanisms such as dynamic step size, learning factors, and annealing temperature enhances the algorithm’s adaptability and robustness.

## 4. Simulation Analysis of Unmanned Ship Heading Control

### 4.1. System Response Under Different Time Delay Conditions

The initial heading angle is set at 0°. For simulation analysis, the unmanned ship’s goal headings are set to 30° and 60°. Wind, waves, currents, and other interferences will all have an impact on the unmanned ship during navigation. White noise and a second-order wave transfer function are employed to approximate these interferences, making the simulation procedure simpler.

**Figure 9:**
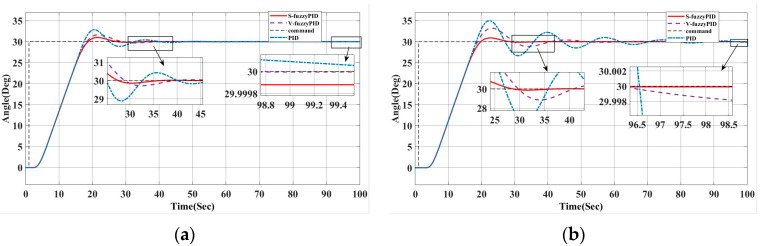
(**a**) Comparison of Smith’s estimated heading under one-second delay; (**b**) Comparison of Smith’s esti-mated heading under a two-second delay condition.

To further validate the control system’s resistance to time delay, this article simulates the system response under various sensor delay situations. Figure 9 displays the heading control results under various delay situations. For a 1 s delay, the system can recover and track the target heading quickly; for a 2 s delay, the system response recovery is delayed, but due to the Smith controller’s compensation effect, the heading tray is effectively controlled, and the response time is increased by only about 10%.

Figure 9 displays the control system’s simulated responses under varying sensor delays, with the detailed results summarized in Table 3 and Table 4. While 1 s and 2 s delays degrade performance, the Smith predictor compensation ensures effective heading control. For these delays, the PID controller achieves shorter rise and settling times but suffers from increased overshoot. By contrast, the V-fuzzyPID and S-fuzzyPID controllers demonstrate enhanced stability and reduced overshoot. Notably, under a 2 s delay, the S-fuzzyPID controller not only exhibits the lowest overshoot but also achieves significantly smaller steady-state error than the other controllers, highlighting its superior delay robustness.

### 4.2. Optimization Effect of Controller

#### 4.2.1. Unmanned Ship Simulation Analysis

To verify the effectiveness of the proposed hybrid optimization algorithm, a comparative study with standard algorithms such as genetic algorithm (GA) and particle swarm optimization (PSO) was conducted under different conditions. Key performance indicators such as response speed, stability, control accuracy, and anti-interference ability were evaluated. The results show that the BAS-HSA-GA method significantly improves the optimization efficiency, reduces overshoot and controller oscillation, shortens the convergence time, and reduces the number of iterations by nearly 30%.

Further simulations were performed to verify the robustness and stability of the controller, and its performance indicators such as settling time, response time, and steady-state error are listed in Table 4. The fuzzy PID controller always maintains low error and reliable system behavior.

**Figure 10:**
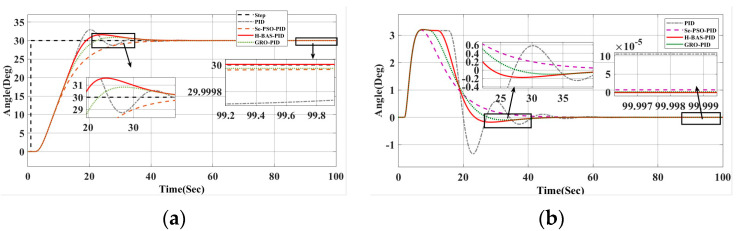
(**a**) Response curve at 30° without interference; (**b**) Curve of heading angle change at 30° without interference.

**Figure 11:**
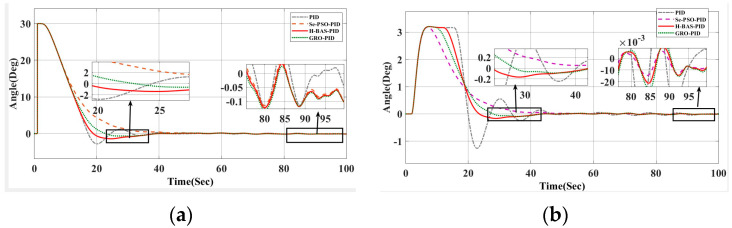
(**a**) Error curve with interference at 30°; (**b**) Heading angle change curve under 30° interference.

Figure 10 and Figure 11 show the changes in the rudder angle and tracking error under the four optimization strategies, respectively. The data summarized in Table 4 and Table 5 correspond to two scenarios: Figure 10 shows the performance without disturbance when the target heading is 30°, while Figure 11 reflects the system behavior under external disturbance. The results confirm that the proposed hybrid algorithm has excellent adaptability and robustness in dynamic and uncertain environments.

The enhanced hybrid beetle antenna search (BAS) algorithm demonstrates superior performance compared to conventional PID, GRO-optimized fuzzy PID, Se-PSO-optimized fuzzy PID, and the Smith predictor-based variable universe fuzzy PID controller. As evidenced by the results in Table 5 and Table 6 and the plots in Figure 10 and Figure 11, the proposed approach yields lower overshoot, faster convergence, and smoother rudder actuation under both nominal and disturbed conditions. This improved stability minimizes rudder activity, thereby reducing mechanical wear and enabling more accurate and efficient course-keeping.

**Figure 12:**
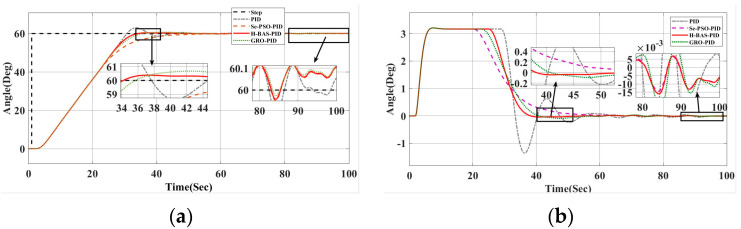
(**a**) Response curve under 60° interference; (**b**) 60° rudder angle change curve under interference conditions.

To further evaluate robustness, the desired heading was adjusted to 60°, and additional simulations were conducted under compound environmental disturbances, including wind, waves, and currents. Key performance indicators—heading accuracy, tracking error, and rudder angle variation—were closely analyzed. As shown in Figure 12 and Table 7, the hybrid BAS algorithm maintained excellent tracking precision and directional stability even in highly dynamic conditions, effectively mitigating external disturbances and ensuring reliable heading control performance.

Figure 12 illustrates the heading angle variation curve under interference from a 60° target heading. The results indicate that the hybrid beetle optimization algorithm outperforms other systems, maintaining the best control performance even with a shift in the target heading. This algorithm demonstrates superior anti-interference capabilities, as shown by its lowest overshoot, fastest response time, and most stable rudder angle change compared to alternative approaches.

Additionally, a Lyapunov stability analysis of the controller, supported by simulation results, confirms that the designed controller ensures the system operates stably. The simulations further validate that the controller effectively reduces error and maintains system stability under interference conditions. These findings suggest that the controller has strong potential for practical applications in unmanned surface vessel heading control, particularly in complex and dynamic environments.

**Figure 13:**
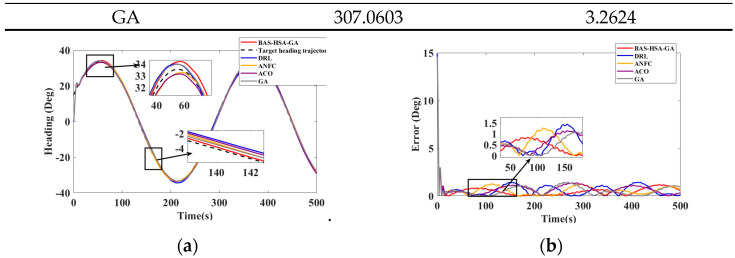
(**a**) S-type path tracking change curve, (**b**) S-type path tracking error curve.

According to the curves in Figure 13, the data in Table 8 show that the BAS-HAS-GA method achieves the best S-type path tracking performance with the lowest IEA (271.6276) and ITEA (3.1021), outperforming DRL, ANFC, ACO, and GA in both accuracy and error reduction. Future research can combine the advantages of BAS-HSA-GA and DRL to develop more efficient path tracking algorithms.

#### 4.2.2. Field Test of Unmanned Surface Vehicle (USV)

**Figure 14:**
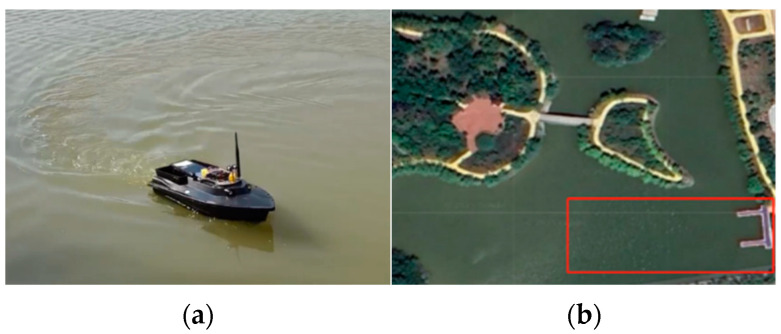
(**a**) Experimental unmanned boat; (**b**) Experimental water environment.

To demonstrate the feasibility of the proposed control strategy, this section details a real test of the heading control of an unmanned surface vessel (USV). The USV is equipped with a series of sensors, a microcontroller, and a host computer to enable real-time data management and vehicle performance supervision. The experiment was conducted in the waters of Jingsi Lake in Lianyungang City. The USV is shown in Figure 14, and the setup is shown in Figure 14b, the red box indicates the navigation range.

**Figure 15:**
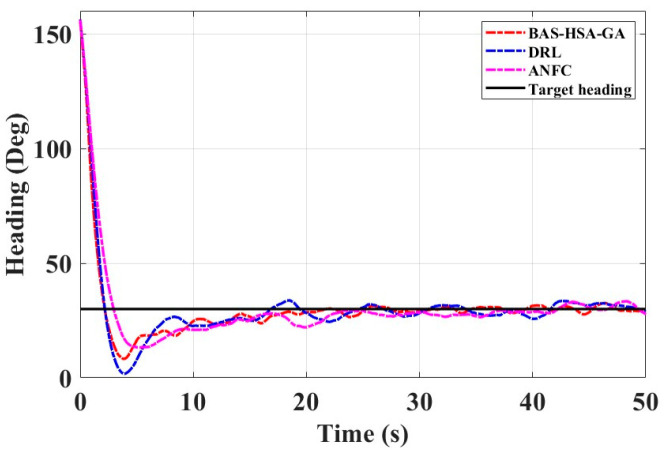
Course change curve of the experimental ship.

The field test was conducted in moderate sea conditions, with sea state level 4 and wind force level 4, providing a challenging but realistic environment for evaluating the robustness and performance of the system. The field test results are shown in Figure 15, including the change in heading angle over time, and Table 9 shows the control performance indicators.

It can be seen from Figure 15 and Table 9 that the control method optimized by the BAS-HSA-GA is better than the deep learning and adaptive fuzzy neural network control methods. The values of the performance indicators IAE and ITAE of the hybrid algorithm are smaller than the values of the performance indicators IAE and ITAE of the other two control strategies.

To further verify the reliability and stability of the experiment, the unmanned boat was subjected to linear motion in unobstructed and undisturbed distant waters and linear obstacle avoidance heading control in disturbed near-view waters. By comparing with other mainstream control algorithms, the advantages of the BAS-HAS-GA in steady-state control and path tracking were evaluated.

**Figure 16:**
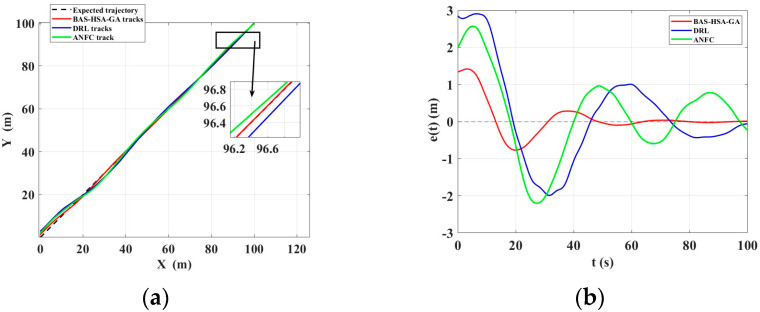
(**a**) Unmanned ship track comparison, (**b**) Steady-state error comparison.

Figure 16 and Table 10 show that the BAS-HAS-GA control method, after using the Smith prediction method, outperforms the other two methods in terms of total error, time-weighted error, and steady-state error, demonstrating its rapid convergence, strong stability, and enhanced disturbance rejection in track tracking.

To test the control capabilities of the unmanned vessel in complex, close-range operating waters, an obstacle environment was set up to test the obstacle avoidance path planning and control stability of the unmanned vessel. When the unmanned vessel’s distance from the obstacle center is less than the obstacle radius + the safety margin, it enters obstacle avoidance mode and adjusts the target heading to an angle offset from the current heading (default 45°). After leaving the obstacle avoidance area, it briefly enters recovery mode, slightly correcting the target heading to return to the original path.

**Figure 17:**
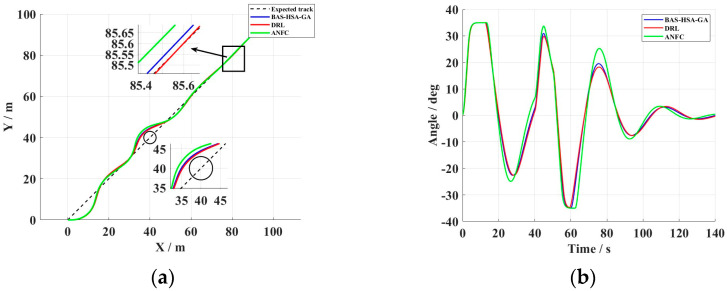
(**a**) Track curve. (**b**) Rudder angle change curve.

The proposed control scheme with an improved Smith predictor demonstrates superior performance in obstacle avoidance tasks, as evidenced by Figure 17 and Table 11. It maintains minimal tracking error under system delays and external disturbances, highlighting its strong delay compensation and real-time adjustment capabilities.

Compared to DRL and ANFC methods, the Smith-based controller excels in heading maintenance, path tracking, and dynamic obstacle avoidance, particularly in suppressing steady-state deviation and enhancing disturbance rejection. Its adaptive time-delay compensation and feedforward–feedback structure effectively address time-varying delays and improve stability, enabling rapid convergence and stable navigation of unmanned vessels in complex waters. This offers an efficient solution for high-precision control of marine unmanned systems.

## 5. Conclusions

This study has successfully developed and validated an advanced, robust heading control system for unmanned surface vessels that effectively addresses the challenges of environmental disturbances and system delays through the integration of an enhanced Smith predictor, a variable-universe fuzzy PID controller with self-adaptive scaling domains, and a hybrid BAS-HSA-GA optimization algorithm. The experimental results demonstrate significant performance improvements, including a 90% reduction in steady-state error and a 30% faster settling time compared to conventional methods, along with 15–25% lower tracking errors in sea-state 4 conditions, while Lyapunov stability analysis confirms the system’s theoretical robustness. However, the current implementation has several limitations that need to be addressed in future research, including its reliance on a first-order Nomoto model that may not fully capture higher-order dynamics, the computational complexity of the hybrid optimization algorithm that could challenge real-time implementation on low-power systems, and the need for testing under more extreme sea conditions beyond sea-state 4. Future work should focus on integrating reinforcement learning techniques for online adaptive tuning, developing simplified optimization variants to reduce computational load, extending the framework to cooperative multi-USV scenarios with distributed control architecture, implementing the system on FPGA hardware platforms for enhanced real-time processing, conducting comprehensive testing under extreme sea conditions (sea-state 5-6), and incorporating model predictive control elements to further improve trajectory tracking performance, which would significantly advance autonomous marine vehicle control capabilities for high-precision navigation in challenging maritime environments.

## Figures and Tables

**Table 1:** Literature Review on Key Control Strategies for USV Heading Control.

Ref.	Authors (Year)	Question	Workaround
[4]	Li et al., (2025)	The performance is limited in nonlinear, coupled and time-delay systems, resulting in reduced control accuracy and stability.	Propose a method that significantly improves PID (e.g., ITAE/output curve comparison) compared to the same optimizer
[5]	Guo et al., (2020)	Under uncertain ocean conditions and external disturbances, the performance of the classic Smith predictor degrades significantly.	Attempts are made to enhance SP adaptability through adaptive or data-driven modifications and integration with intelligent controllers.
[6]	Sariyildiz (2025)	The above improved methods are difficult to balance compensation accuracy and real-time robustness in highly dynamic environments.	Introducing a unified state space architecture, compatible with ZO/HO DOb, and supporting HP-DOb, and introducing a unified state space architecture, compatible with ZO/HO DOb, and supporting HP-DOb
[7]	Cao et al., (2016)	The domain of traditional fuzzy PID is usually fixed and difficult to adapt to error changes under different working conditions.	Variable universe fuzzy control (VUFC) can adaptively adjust the input and output universe according to the error size.
[8]	Lu et al., (2016)	Although VUFC exhibits better dynamic performance in nonlinear and time-varying systems, its parameter adjustment still relies on experience and is difficult to achieve global optimization.	The existing problems of the VUFC method are pointed out, which leads to the motivation of using optimization algorithm for parameter tuning in this paper.

**Table 2:** Unmanned ship model data.

Boat Length/m	Boat Width/m	Boat Speed/kn	Full Load Draft/m	Full Load Displacement/m^3^	Block Coefficient
7.02	2.06	35	0.32	2.73	0.6976

**Table 3:** Comparison of different Smith controllers under one-second delay condition.

Control Method	Rise Time/s	Stabilization Time/s	Overshoot	Error/10^−6^
PID	20.3	45.9	9.7%	3.077
V-fuzzyPID	22.8	43.3	2.6%	24.94
S-fuzzyPID	26.8	38.1	0.3%	119.5

**Table 4:** Comparison of different Smith controllers under two-second delay conditions.

Control Method	Rise Time/s	Stabilization Time/s	Overshoot	Error/10^−6^
PID	22.4	87.9	16.7%	1278
V-fuzzyPID	23.6	57.1	8.5%	2218
S-fuzzyPID	28.5	37.8	0.3%	83.13

**Table 5:** Comparison of heading control optimized by different algorithms.

Control Method	Rise Time/s	Stabilization Time/s	Overshoot	Error/10^−6^
PID	20.3	74.5	9.7%	258.9
H-BAS-PID	22.9	70.5	5.7%	33.71
GRO-PID	28.2	76.5	2.1%	3.319
Se-PSO-PID	42.2	77.7	0	26.01

**Table 6:** Rudder performance indicators of BAS-HSA-GA and other control algorithms at a heading angle of 30°.

Control Method	Rudder Turning Time/s	Stabilization Time/s	Stable Rudder Angle
PID	15.67	56.866	0.1109
Se-PSO-PID	6.866	61.137	0.03717
H-BAS-PID	13.042	42.573	0.04619
GRO-PID	10.414	47.813	0.05379

**Table 7:** Rudder performance indicators of BAS-HSA-GA and other control algorithms at a heading angle of 90°.

Control Method	Rudder Turning Time/s	Stabilization Time/s	Stable Rudder Angle
PID	42.214	76.183	0.1072
Se-PSO-PID	34.133	73.75	0.02175
H-BAS-PID	38.535	70.598	0.01468
GRO-PID	36.498	73.292	0.02872

**Table 8:** Curve path tracking indicators.

Control Methods	IEA	ITEA
BAS-HAS-GA	271.6276	3.1021
DRL	336.7751	3.5838
ANFC	304.3595	3.2522
ACO	360.4539	3.2120

**Table 9:** Heading control performance indicators.

Control Methods	IEA	ITEA
BAS-HSA-GA	305.91	2254.62
DRL	359.87	3304.06
ANFC	382.71	3564.76

**Table 10:** BAS-HAS-GA optimal straight line track performance indicators.

Control Methods	IEA	ITEA	Steady-State Error
BAS-HAS-GA	38.0956	787.3398	0.0119
DRL	100.8489	2915.8468	0.0343
ANFC	96.0351	3134.4509	0.2207

**Table 11:** Performance indicators of BAS-HAS-GA optimal straight line obstacle avoidance trajectory.

Control Methods	IEA	ITEA	Steady-State Error
BAS-HAS-GA	2045.94	775,911.43	0.05
DRL	2102.06	811,113.60	0.33
ANFC	2100.02	818,123.40	0.46

## Data Availability

The datasets generated and analyzed during the current study are available from the corresponding author on reasonable request.

## Abbreviations

**Symbol**	**Description**	**Unit**
e(t)	System error at time t	
$\dot{e(t)}$	Derivative of the error	
$U_{x0}$	Initial input universe of discourse	
$U_{y0}$	Initial output universe of discourse	
$k_{x}(t)$	Dynamic scaling factor for input universe	
$k_{y}(t)$	Dynamic scaling factor for output universe	
$\alpha_{x},\alpha_{y}$	Tuning coefficients for scaling factors	
β,γ	Exponents for nonlinear transformation	
$E_{max}$	Maximum error value	
α(t)	General time-varying scaling factor	
p	Power exponent for scaling factor	
$G_{c}(s)$	Transfer function of the controller	
$G_{p}(s)$	Transfer function of the plant (without delay)	
$G_{m}(s)$	Transfer function of the estimation model	
τ	Actual time delay of the plant	s
$\tau_{m}$	Time delay of the estimation model	s
r(s)	System input (reference signal)	
y(s)	System output	
${\overset{ˇ}{y}}_{\psi }$	Predicted system output	
$G_{\psi }(s)$	Heading transfer function of the USV	
$K_{\psi }$	Heading gain	s
$\tau_{\psi }$	Heading time constant	s
K	Nomoto model gain (identified)	
T	Nomoto model time constant (identified)	s
$Y_{d}(s)$	Wave disturbance output	
ω(s)	Zero-mean Gaussian white noise	
h(s)	Second-order wave transfer function	
$K_{\omega }$	Wave model gain	
ζ	Damping ratio	
$\omega_{0}$	Dominant wave frequency	rad/s
$\sigma_{\omega }$	Wave intensity coefficient	
$T_{\omega }$	Characteristic wave period	s
$h_{1/3}$	Significant wave height	m
